# Validation of prognostic and predictive value of total tumoral load after primary systemic therapy in breast cancer using OSNA assay

**DOI:** 10.1007/s12094-023-03347-7

**Published:** 2023-12-09

**Authors:** Laia Bernet-Vegué, Carolina Cantero-González, Magdalena Sancho de Salas, David Parada, Tiziana Perin, Zulma Quintero-Niño, Begoña Vieites Pérez-Quintela, Douglas Sánchez-Guzmán, Marina Castelvetere, David Hardisson Hernaez, María Dolores Martín-Salvago

**Affiliations:** 1 Breast Area, Department of Anatomic Pathology, Ribera Salud Hospitals, Valencia, Spain; 2 Department of Pathology, Complejo Hospitalario de Jaen, Jaén, Spain; 3 Departamento de Anatomía Patológica del, Complejo Asistencial Universitario de Salamanca, Salamanca, Spain; 4 Molecular Pathology Unit, Department of Pathology, Hospital Universitari de Sant Joan, Institut d’Investigació Sanitària Pere Virgili, Facultat de Medicina i Ciències de la Salut, Universitat Rovira i Virgili, Reus, Tarragona Spain; 5 Pathology Unit, Centro di Riferimento Oncologico di Aviano (C.R.O.), IRCCS, Aviano, Italy; 6 Departamento de Anatomía Patológica, Hospital Universitario La Ribera, Alzira, Spain; 7 Department of Pathology, University Hospital Virgen del Rocío, Seville, Spain; 8 Pathology Department, Arnau de Vilanova University Hospital, Lleida, Spain; 9 Pathological Anatomy Laboratory, Casa Sollievo della Sofferenza, San Giovanni Rotondo, FG Italy; 10 Department of Pathology, Hospital Universitario La Paz, Molecular Pathology and Therapeutic Targets Group, Hospital La Paz Insitute of Research (IdiPAZ), Center for Biomedical Research in the Cancer Network (CIBERONC), Instituto de Salud Carlos III, Faculty of Medicine, Universidad Autónoma de Madrid, Madrid, Spain

**Keywords:** Breast cancer, Axillary lymphadenectomy, Total tumor load, Sentinel lymph node biopsy, Primary systemic treatment

## Abstract

**Purpose:**

This study aimed to validate the classification of breast cancer (BC) patients in progression risk groups based on total tumor load (TTL) value to predict lymph node (LN) affectation after neo-adjuvant systemic therapy (NAST) obtained in the NEOVATTL study.

**Methods/patients:**

This was an observational, retrospective, international, multicenter study including patients with infiltrating BC who received NAST followed by sentinel lymph node biopsy (SLNB) analyzed with one-step nucleic acid amplification (OSNA) from nine Spanish and two Italian hospitals. Patients were classified into three groups according to the progression risk, measured as disease-free survival (DFS), based on TTL values (> 250, 250–25,000, and > 25,000 copies/μL). The previous (NEOVATTL study) Cox regression model for prognosis was validated using prognostic index (PI) and Log ratio test (LRT) analyses; the value of TTL for axillary non-SLN affectation was assessed using receiver operating characteristic (ROC) curves.

**Results:**

We included 263 patients with a mean age of 51.4 (± SD 10.5) years. Patients with TTL > 25,000 copies/μL had a shorter DFS (HR 3.561 [95% CI 1.693−7.489], *p* = 0.0008 vs. TTL ≤ 25,000). PI and LRT analyses showed no differences between the two cohorts (*p* = 0.2553 and *p* = 0.226, respectively). ROC analysis showed concordance between TTL and non-SLN involvement (area under the curve 0.828), with 95.7% sensitivity and 92.9% specificity at a TTL cut-off of > 15,000 copies/μL.

**Conclusions:**

In BC patients who had received NAST and underwent SLNB analysis using OSNA, a TTL value of > 25,000 copies/μL was associated with a higher progression risk and > 15,000 copies/μL was predictive of non-SLN involvement.

**Supplementary Information:**

The online version contains supplementary material available at 10.1007/s12094-023-03347-7.

## Introduction

Neo-adjuvant systemic treatment (NAST) is currently being used as a preoperative treatment modality in early-stage breast cancer (BC), offering the opportunity to further de-escalate the surgical management of the axilla in patients undergoing BC surgery [1–4]. However, there is still a lack of consensus regarding the post-treatment surgical approach to axillary lymph node dissection (ALND).

Sentinel lymph node biopsy (SLNB) has been established as the gold standard for pathologic evaluation of the axilla in patients with operable BC [5]. However, attitudes upon finding metastatic disease in sentinel lymph nodes (SLN) post-NAST vary widely in clinical practice, with a trend toward a conservative approach. Currently, international consensuses recommend lymphadenectomy for any type of metastatic involvement and many clinical guidelines still lack recommendations for post-NAST SLN [6–8].

The diagnostic performance of SLNB by conventional histopathology after NAST remains controversial as this treatment could limit the accuracy of the nodal histological evaluation [9–11]. Cytokeratin 19 (CK19) is a membrane protein expressed by most BC even post-NAST, constituting a good biomarker to detect metastases [12, 13]. One-step nucleic acid amplification (OSNA) is a molecular technique that detects the messenger ribonucleic acid (mRNA) copy number of CK19 present in SLN. OSNA enables calculating the total tumor burden (TTL) in an automated and reproducible manner, providing objective information about the metastatic burden present in lymph nodes (LNs) [14], detecting micro- and macro-metastases of LNs with high sensitivity and specificity [15, 16]. Therefore, its use could provide more sensitive information regarding the LNs metastatic status post-NAST compared to conventional histological examination, limiting overtreatment or unnecessary ALND and adding more precision and reproducibility to axillary surgical management [9, 17].

The PLUTTO study demonstrated TTL's clinical value in BC patients without NAST, correlated TTL with disease-free survival (DFS), and identified low- and high-risk progression groups based on a TTL cut-off of 25,000 copies/μL [18]. Piñero-Madrona et al. [19] validated the prognostic value of TTL in 5-year DFS proposed in the PLUTTO study. The NEOVATTL study demonstrated the prognostic value of TTL measured by OSNA in BC patients post-NAST, showing a clear decrease in survival at TTL ≥ 25,000 copies/μL [20]. Moreover, a prognostic scoring tool that considered TTL, Ki67 score before NAST, and treatment response in the primary tumor (Miller–Payne grade) developed in the NEOVATLL study found decreased DFS in patients with a high TTL (> 25,000 copies/μL), high Ki67 (> 20%), or low Miller–Payne grade (1 or 2).

This retrospective study aimed to validate the classification of patients in progression risk groups based on TTL values and the predictive value of TTL regarding LN affectation obtained in the NEOVATTL study in a multi-centric cohort. Additionally, we aimed to establish correlations between TTL values and the risk of positive LNs.

## Materials and methods

### Study design and population

This was an observational, retrospective, international, multicenter study including patients with infiltrating BC. Patients’ electronic medical records (EMR) were obtained from nine Spanish and two Italian hospitals. Patients who received NAST followed by SLNB with OSNA before December 2015 were included in the study. The SLNB was performed according to each center’s protocol, using Tc99 and/or blue dye to map the SLN. Patients under 18 years of age, with carcinoma in situ or other malignant neoplasms, those considered by the investigator to be unsuitable for the study, and those who underwent OSNA in 2012 and earlier and were included in the NEOVATTL study were excluded.

Primary data recorded in EMR were collected between June and July 2019. Data were anonymized at the center of origin in compliance with Organic Law 3/2018 of December 5 on the Protection of Personal Data and guarantee of digital rights [21]. This study was performed after obtaining approval by the Comité Ético de Investigación Clínica del Departamento de Salud de La Ribera (Clinical Research Ethics Committee of La Ribera Health Department). Furthermore, it was developed following the ethical principles originating from the latest version of the Declaration of Helsinki accepted by local authorities and which are in line with Good Clinical Practice (GCP) and the requirements of current Spanish regulations.

### Endpoints and variables

The main objective of this study was to validate the classification system for prognosis, in terms of DFS, based on TTL values (according to the risk of progression TTL > 250, TTL 250−25,000, and TTL > 25,000 CK19 mRNA copies/μL), and the scoring tool (TTL + Ki67 + Miller–Payne) developed in the NEOVATTL study. As a secondary objective, this study aimed to validate the results obtained in the NEOVATTL study regarding the use of TTL values to predict non-SLN involvement.

Demographic (i.e., age), surgical, and pathologic tumor characteristics before and after NAST were collected from medical records. Surgery characteristics included data related to SLNB (TTL) and ALND (yes/no; metastatic nodes/non-sentinel nodes removed). Tumor characteristics included histological type, grade, hormonal receptors status (i.e., estrogen and progesterone receptors), and molecular subtype pre- and post-NAST. The date of the last follow-up visit and disease progression (with date) were additionally recorded.

Details on study definitions, including TTL, DFS, and prognostic scores are provided in the supplementary materials file.

### Statistical analysis

A descriptive analysis of all the variables of this and the previous study (NEOVATTL) was performed by study and on pooled data from both studies. Quantitative variables were described using the mean and the standard deviation (SD) and qualitative variables were described as absolute and relative frequencies with the 95% confidence interval (95% CI). The study populations were compared using Fisher’s exact, Pearson chi-squared, and Mann–Whitney tests.

The primary validation purpose focused on applying the multivariate Cox regression model built in the NEOVATTL study to the data of a validation cohort. The variables included in the model were TTL with a cut-off point of 25,000 copies/μL, the cell proliferation index Ki67 with a cut-off point of 20%, and the Miller–Payne classification.

The framework established by Royston and Altman was followed to perform a rigorous validation [22], which initially involved the execution of a calibration test. This calibration test assesses the concordance between the survival probabilities predicted by the initial model and those observed in the validation data of this study, with adequate concordance being an indicator of successful calibration and thus providing confidence in the model's predictions.

A crucial aspect at this stage was the calculation of the Prognostic Index (PI) or calibration slope, a weighted sum of the model variables using the regression coefficients estimated by the Cox model. The PI is crucial in validation, providing a quantitative risk metric regarding calibration and discrimination in the validation data set. Additionally, we investigated whether the PI was statistically different from 1, indicating a difference in discrimination between the original and validation data sets: a PI < 1 may indicate that the validation model underestimates the risk in certain cases; contrarily, a PI > 1 may indicate that the validation model overestimates the risk. In both scenarios, a PI different from one indicates that the model's discrimination is less accurate in the validation data set compared to the original data set.

The final validation stage focused on applying the Likelihood Ratio Test (LRT) to compare the Hazard Ratios (HR) between cohorts in the models derived from the original and validation data sets. The LRT provides statistical evidence of consistency or discrepancy in the risk coefficients between the two models. Furthermore, the stratification in four risk groups using the scoring system developed in the NEOVATTL study was validated using PI calibration and the LRT comparing the HR between the two models.

For the secondary objective, areas under the receiver operating characteristic (ROC) curve (AUC) were determined to evaluate the predictive value of TTL for axillary non-SLN involvement in the validation cohort. The sensitivity, specificity, positive predictive value (PPV), and negative predictive value (NPV) of TTL were evaluated at different cut-off points. The probability of finding positive ALND according to the cut-off points was evaluated by dividing false negatives by a sum of false negatives and true negatives.

The sample size was estimated at 253 patients. This estimate was based on a multivariate Cox regression that allows us to detect a change in the recurrence rate (12.4% according to the NEOVATTL study) with a hazard ratio of 2.96 for the adjusted TTL model, assuming a standard deviation for this variable of 0.5348 (calculated based on the results of the NEOVATTL study) and a moderate correlation (0.2524) with the rest of the variables included in the model. This patient number allowed conclusions to be derived with 80% statistical power and an alpha error of 5%. All analyses were performed using R software version 4.2.1.

## Results

### Characteristics of the study population

A total of 263 patients were included in this validation study. Additionally, data from this validation cohort and previous data from the NEOVATTL study were analyzed together, resulting in 577 patients for pooled analyses. Those patients with SLNB between 2012 and 2015 were included, and therefore, follow-up times until the data collection in 2019 might vary. The mean age of the patients in this study was 51.4 (± SD 10.5) years. Pathologic tumor characteristics pre- and post-NAST in both cohorts are described in Table S2.

### TTL and surgery characteristics

Most patients had TTL values < 25,000 CK19 mRNA copies/μL with no significant differences between cohorts (Table 1). Regarding the characteristics of the surgery, summarized in Table 2, approximately 60% of patients did not undergo ALND with no significant differences between cohorts. In both cohorts, most patients had 1−2 SLN dissected for biopsy, even though a higher proportion of patients in the NEOVATTL cohort had > 2 SLN (*p* < 0.0001). Around 70% of patients had < 9 non-SLN removed, with no significant differences between studies. Most patients in the validation cohort lacked metastatic SLN compromise in the SLNB post-NAST, whereas most patients in the NEOVATLL cohort had 1−3 compromised SLN (*p* < 0.0001). The NEOVATTL Validation study included a completely different population recruited in different centers, likely explaining this difference between cohorts (Table 2).

**Table 1:** Total tumor load (copies/µL)

	Total (*N* = 577)	NeovaTTL (*N* = 314)	NeovaTTL Validation (*N* = 263)	*P*-value
Mean (SD)	54233, 4 (421610, 6)	42313, 8 (248208, 3)	68464, 3 (562865, 5)	0.5025^a^
95%CI	(19832, 3; 88634, 4)	(14860, 2; 69767, 4)	(438, 3; 136490, 3)	
*TTL value categories, n (%)*				
TTL < 250	362 (62.7)	196 (62.4)	166 (63.1)	0.93^b^
TTL 250−25,000	151 (26.2)	84 (26.8)	67 (25.5)	
TTL > 25,000	64 (11.1)	34 (10.8)	30 (11.4)	

SD, standard deviation; 95%CI, 95% confidence interval; TTL, total tumor load

^a^Mann–Whitney test comparing both cohorts

^b^Pearson chi-squared test comparing both cohorts

**Table 2:** Surgery Characteristics, n (%)

	Total	NeovaTTL	NeovaTTL Validation	*P*-value
*ALND*				
N	577	314	263	
No	363 (62.9)	187 (59.6)	176 (66.9)	0.0700^a^
Yes	214 (37.1)	127 (40.4)	87 (33.1)	
*N° SLN removed (SLNB post-NAST)*				
N	577	314	263	
1−2	464 (80.4)	227 (72.3)	237 (90.1)	< 0.0001^a^
> 2	113 (19.6)	87 (27.7)	26 (9.9)	
*N° non-SLN removed*				
N	564	314	250	
< 9	405 (71.8)	219 (69.7)	186 (74.4)	0.2583^a^
≥ 9	159 (28.2)	95 (30.3)	64 (25.6)	
*N° Metastatic SLN (SLNB post-NAST)*				
N	387	124	263	
0	164 (42.4)	0 (0.0)	164 (62.4)	< 0.0001^b^
1−3	215 (55.6)	117 (94.4)	98 (37.3)	
> 3	8 (2.1)	7 (5.6)	1 (0.4)	

*SLN*, Sentinel lymph node; *NAST*, Neo-adjuvant systemic treatment; *ALND*, axillary lymph node dissection

^a^Fisher’s exact test comparing both studies

^b^Pearson chi-squared test comparing both studies

### TTL value as prognostic factor for progression

**Fig. 1:**
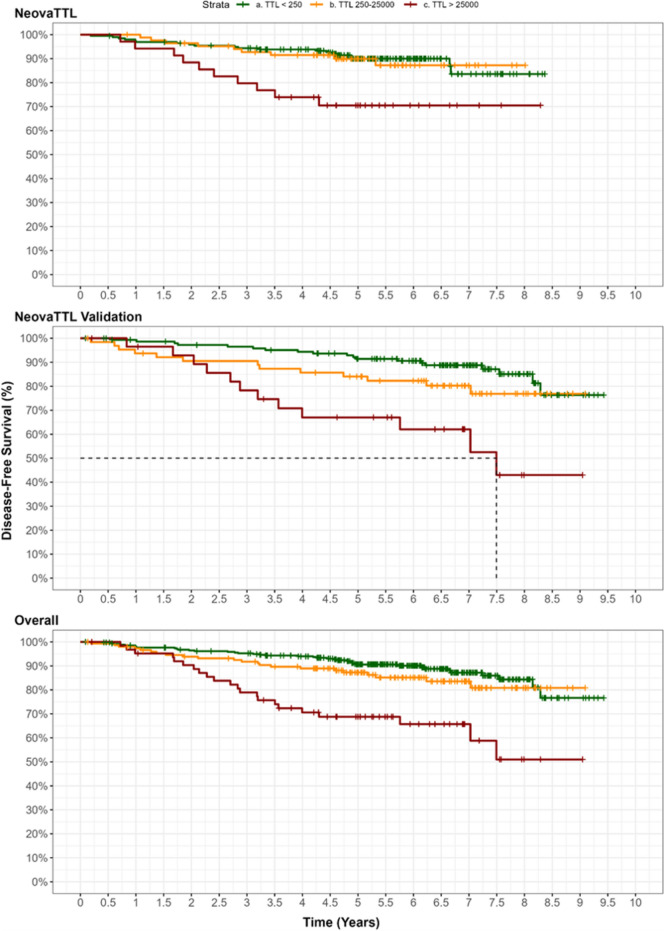
Kaplan–Meier estimates of disease-free survival according to total tumor load (TTL) cut-off points. **A** NEOVATTL study. **B** NEOVATTL Validation **C** Overall

The Kaplan–Meier curves evaluating time to disease progression in three patient groups according to TTL values showed that patients with TTL > 25,000 copies/μL had a shorter DFS (Fig. 1). DFS at 5 years in patients with TTL > 25,000 copies/μL was 68.5% (95% CI 57.7−81.3%) years, compared to 90.6% (95% CI 87.4−93.8%) and 87.2% (95% CI 81.9−92.9%) years in patients with TTL < 250 and TTL 250–25,000 copies/μL, respectively. Kaplan–Meier curves were similar in both cohorts analyzed separately and jointly, confirming the value of TTL > 25,000 copies/μL as cut-off for disease prognosis (Fig. 1).

The validation cohort was stratified on risk levels according to three prognostic factors identified in the NEOVATTL: TTL, Ki67, and Miller–Payne. HR for disease progression obtained in the multivariate Cox regression model according to TTL (> 25,000 vs. ≤ 25,000 copies/μL) and Ki67 (> 20% vs. ≤ 20%) were similar and statistically significant in both cohorts, showing increased risk of progression in patients with TTL > 25,000 copies/μL (HR 3.561 and 3.665 in NEOVATTL and validation cohorts, respectively) and those with Ki67 > 20% (HR 4.131 and 2.001 in NEOVATTL and validation cohorts, respectively) (Table 3). HR values for Miller–Payne grades were lower compared to those for TTL values in both studies (HR 1.407 and 1.310 in NEOVATTL and validation cohorts, respectively) with no statistical significance in the validation cohort.

**Table 3:** Prognostic factors for disease-free survival in the NEOVATTL and validation cohorts

	HR	95% CI low	95% CI up	*P*-value
*NEOVATTL cohort, n* = *298*				
TTL > 25,000 vs TTL ≤ 25,000	3.561	1.693	7.489	0.0008
Ki67 PRE* > 20% vs ≤ 20%	4.131	1.853	9.207	0.0005
Miller−Payne (REV)**	1.407	1.075	1.842	0.0130
*NEOVATTL Validation cohort, n* = *231*				
TTL > 25,000 vs TTL ≤ 25,000	3.665	1.673	8.028	0.0012
Ki67 PRE* > 20% vs ≤ 20%	2.001	1.011	3.960	0.0463*
Miller−Payne (REV)**	1.310	0.972	1.765	0.0759

*DFS*, disease-free survival; *HR*, hazard ratio; *95%CI*, 95% confidence interval; *Low*, lower bound; *Up*, Upper bound; *TTL*, total tumor load

*Before neo-adjuvant systemic therapy

**Revised version of Miller–Payne

PI analysis of the multivariate Cox models for the TTL, Ki67, and Miller–Payne variables yielded a PI coefficient of 0.783 (95% CI 0.409–0.191, *p* < 0.0001), as shown in Figure S1. This coefficient was close to 1 (− 0.22, 95% CI − 0.591–0.157) and not significantly different from 1 (*p* = 0.2553), suggesting a congruence between the predictions made by the original NEOVATTL model and those observed in the validation cohort.

Additionally, the HR obtained in the NEOVATTL and validation cohorts for all prognostic factors using the LRT were compared, yielding no statistically significant differences (*p* = 0.226) between the two multivariate models (Table S4).

### Scoring system value as prognostic factor for progression

Kaplan–Meier curves evaluating DFS according to prognostic score were similar in both cohorts analyzed separately and jointly, showing a clear difference in risk of progression in those patients with a score of 4 (Figure S2).

PI analysis of the multivariate Cox models for the prognostic score variables yielded a PI coefficient of 0.738 (95% CI 0.385−1.091; *p* < 0.0001), as depicted in Figure S3. This coefficient is close to 1 (− 0.26, 95% CI 0.615 – 0.091) and not significantly different from 1 (*p* = 0.1454), suggesting congruence between the predictions made by the original NEOVATTL model and those observed in the validation cohort. Additionally, the HR obtained in the NEOVATTL and validation cohorts for all prognostic factors using the LRT were compared, yielding no statistically significant differences (*p* = 0.2) between the two multivariate models (Table S4).

### TTL values as predictive factor for LN involvement

**Fig. 2:**
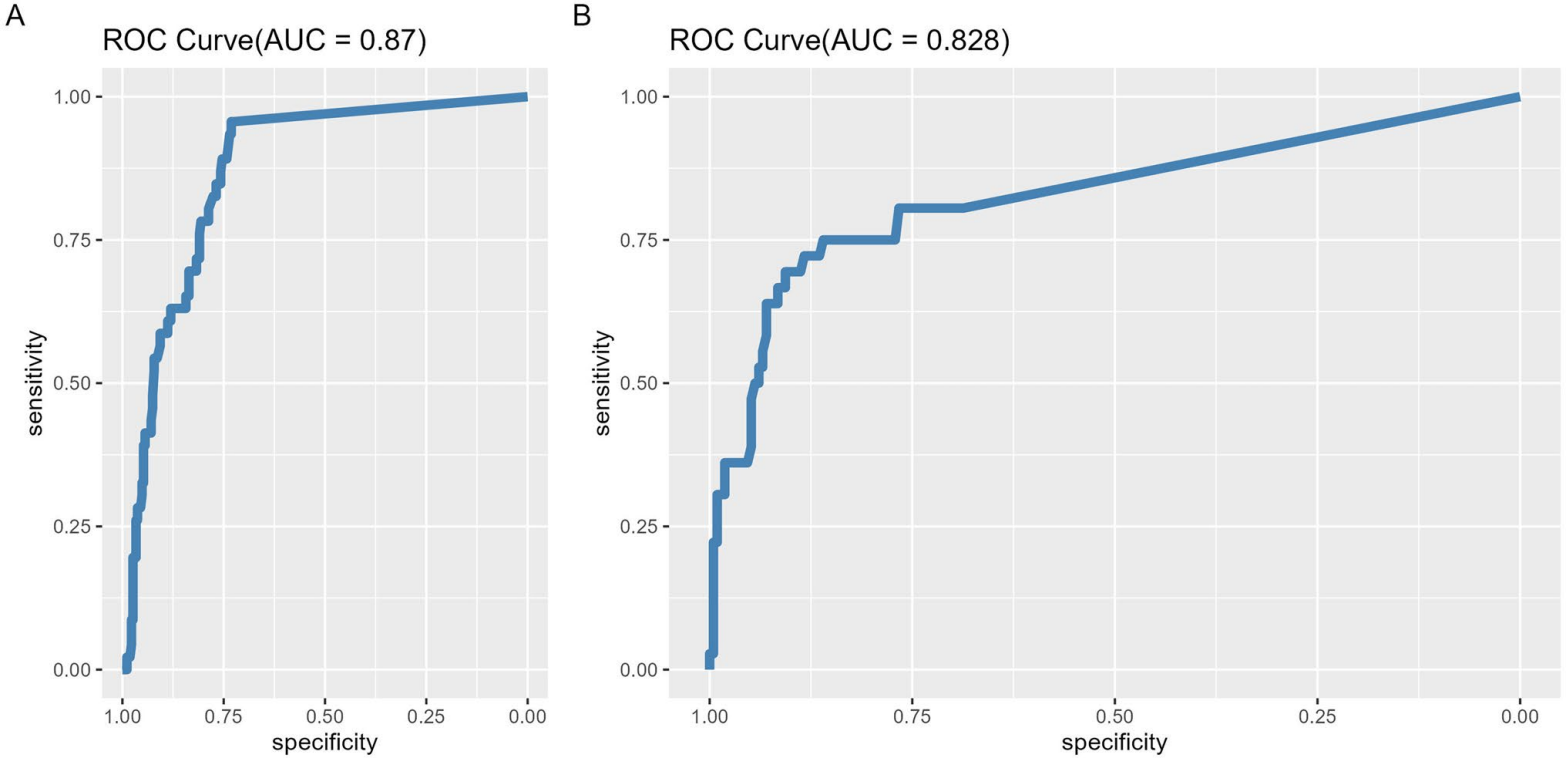
ROC curve based on the sensitivity and the specificity of TTL for the prediction of non-SNL involvement. **A** NEOVATTL study. **B** NEOVATLL Validation. ROC, receiver operator characteristic; SNL, sentinel lymph node; TTL, total tumor load

The ROC curve analysis in the NEOVATTL study demonstrated concordance between TTL and non-SLN involvement, with an AUC of 0.87 (Fig. 2A). At a TTL cut-off of > 15,000 copies/μL, the sensitivity was 95.7%, the specificity 92.9%, with PPV of 51.3% and NPV of 90.5% (Table S5). ROC analysis using data from the validation cohort showed similar results regarding the concordance between TTL and non-SLN involvement, with an AUC of 0.828 (Fig. 2B). At a TTL cut-off of > 15,000 copies/μL, the sensitivity was 58.3%, the specificity 93%, with PPV of 58.3%, and NPV of 93% (Table S5). The probability of finding positive ALND according to the cut-off points was 4.54%, 7%, and 8.2% for the 250, 15,000, and 25,000 copies/μL cut-offs, respectively.

## Discussion

This retrospective study of patients with infiltrating BC (who received NAST followed by SLNB with OSNA) validated the findings of the NEOVATTL study using the same TTL cut-off points to assess the risk of disease progression and DFS (< 250, 250−25,000, and TTL > 25,000 copies/μL), confirming that DFS was significantly shorter among patients with TTL > 25,000 copies/μL than patients with TTL ≤ 25,000 copies/μL. The classification system for prognosis (i.e., risk of progression) based on TTL values, and the scoring tool (TTL + Ki67 + Miller–Payne) obtained in the NEOVATTL study was also validated. A TTL value > 15,000 copies/μL also predicted non-SLN involvement in ALND.

Several histopathologic systems have been described to estimate residual disease and to stratify the prognosis in patients undergoing NAST for BC [23]. Two of the most used scores are the Miller–Payne grading system and the Residual Cancer Burden (RCB) index, which rely on histopathological analysis. Both scores demonstrated to be time-consuming and subjective [22, 23]. On the other hand, Ki67 is a cell proliferation biomarker and its expression in BC has been associated with a worse prognosis and a better response to chemotherapy [24]. The SLNB has been accepted as a good tool for establishing the axillary status in patients with and without NAST, aiming to avoid unnecessary ALND, determine prognosis, and guide treatment decisions. However, the histological assay in SLN is limited due to tissue alterations resulting from the previous systemic treatment in patients receiving NAST and the impossibility of studying the entire lymph node [11, 15]. Furthermore, histological changes post-NAST lack an impact on prognosis and staging according to the latest edition of the American Joint Committee on Cancer (AJCC) [25]. In this regard, OSNA analysis, as a molecular method, may avoid these tissue-associated limitations and provide a highly sensitive and specific quantitative result for the detection of micro- and macro-metastases of LNs compared to conventional histological examination [9, 17].

The NEOVATTL study developed a prognostic scoring tool for DFS, which included TTL as a molecular biomarker, the Ki67 score before NAST, and Miller–Payne grade. Based on this score, DFS decreased with a high TTL (≥ 25,000 copies/μL), high Ki67 (> 20%), or low Miller–Payne grade (Grade 1 or 2). In this study, these prognostic factors were also evaluated independently and combined as a prognostic scoring tool. Although the factors were overall validated, the Miller–Payne classification system showed a lower prognostic value compared to TTL values (decreased HR with no consistent statistical significance). These results suggest a superior prognostic value for TTL. Moreover, the prognostic score was validated showing a clear difference in risk of progression in those patients with a score of 4 (TTL of ≥ 25,000 copies/μL and a Ki67 of > 20%). Altogether, we further validated the proposal of Vieites et al. in the NEOVATTL study [20], regarding the value of this score for disease prognosis in BC patients after ALND.

Although ALND has been associated with postoperative complications and significant morbidity, the surgical strategy for the axillary approach, based on the results of the SLN study, continues to vary widely in clinical practice [26, 27]. This study also validated the results of the NEOVATTL study in which a cut-off of > 15,000 copies/μL TTL was found to be predictive of non-SLN involvement, reinforcing the value of this parameter. These results suggest that restricting ALND to those patients with TTL > 15,000 copies/μL is a reliable approximation for limiting overtreatment or unnecessary ALND and adding more precision to axillary surgical management.

Conventionally, prognostic risk assessment and axillary approach decisions for BC patients have been based on histopathologic methods and criteria. However, the application of these methods is limited due to their low sensitivity, especially for the detection of micro-metastases [28–30]. In this regard, the OSNA method has demonstrated high concordance with histopathological methods with high sensitivity, specificity, and negative predictive value. Moreover, the OSNA assay is a molecular method performed by an automated procedure, with clear advantages in terms of standardization, reproducibility, and objectivity that detects minimal tumor loads that are not always possible to detect with histological methods after NAST. The NEOVATTL study robustly demonstrated the prognostic and predictive value of TTL values assessed by OSNA assay in BC patients undergoing NAST [20]. In the era of individualized treatments, sensitive and specific tools are needed to establish disease prognosis and predict LN involvement. Our study was able to validate with a large sample of patients the results of NEOVATTL, confirming the usefulness of the OSNA method in the classification of patients in different degrees of risk of disease progression and the probability of DFS in addition to the prediction of non-SLN involvement in ALND. The limitations of this study are attributable to the retrospective nature of its design, including the risk of patient selection bias. There were also differences in patient follow-up times between studies but in both studies all patients had at least 5 years of follow-up.

Despite these limitations, the results of this study add evidence that could be relevant to developing protocols using TTL cut-off points to evaluate the risk of progression and to guide individualized surgical strategies regarding the axillary approach in BC patients who have received NAST positioning OSNA assay as a decisive tool for therapeutic de-escalation in post-neo-adjuvant treatment. These results may also increase interest in the generation of prospective, multicenter, randomized, controlled studies to determine the predictive value of TTL for the diagnosis of non-SLN metastases and to establish cut-off points that can guide more conservative surgical strategies in terms of the axillary approach after NAST in patients with BC.

## Conclusion

This study externally validated the NEOVATTL model in a different multi-centric cohort. In BC patients receiving NAST and undergoing OSNA-based SLNB analysis, a TTL > 25,000 copies/μL indicated higher disease recurrence risk, while TTL > 15,000 copies/μL predicted non-SLN involvement. These findings support ALND restriction to TTL > 15,000 copies/μL as a reliable approach, minimizing unnecessary morbidity.

### Supplementary Information

Below is the link to the electronic supplementary material.

Supplementary file1 (DOCX 530 KB)

## Data Availability

The datasets generated during and/or analyzed during the current study are not publicly available but are available from the corresponding author on reasonable request.
