# Supplementary material for: Validation of prognostic and predictive value of total tumoral load after primary systemic therapy in breast cancer using OSNA assay
Source: Clin Transl Oncol. 2023 Dec 9;26(5):1220–8. doi: 10.1007/s12094-023-03347-7 (PMC11026238; doi:10.1007/s12094-023-03347-7)
Supplement: Supplementary file 1 — Supplementary file1 (DOCX 530 KB) [file 12094_2023_3347_MOESM1_ESM.docx]

# Validation of prognostic and predictive value of total tumoral load after primary systemic therapy in breast cancer using OSNA assay

**Journal: Clinical and Translational Oncology**

Authors: Laia Bernet-Vegué^1^, Carolina Cantero-González^2^, Magdalena Sancho de Salas^3^, David Parada^4^, Tiziana Perin^5^, Zulma Quintero-Niño^6^, Begoña Vieites^7^, Douglas Sánchez-Guzmán^8^, Marina Castelvetere^9^, David Hardisson-Hernaez^10^, María Dolores Martín-Salvago^2^.

Affiliations:

^1^Breast Area. Ribera Salud Hospitals. Valencia, Spain.

^2^Department of Pathology. Complejo Hospitalario de Jaen. Jaen, Spain.

^3^Departamento de Anatomía Patológica del Complejo Asistencial Universitario de Salamanca. Salamanca, Spain.

^4^Molecular Pathology Unit. Department of Pathology, Hospital Universitari de Sant Joan. Institut d’Investigació Sanitària Pere Virgili. Facultat de Medicina i Ciències de la Salut, Universitat Rovira i Virgili. Reus (Tarragona), Spain.

^5^Pathology Unit, Centro di Riferimento Oncologico di Aviano (C.R.O.). IRCCS. Aviano, Italy.

^6^Departamento de Anatomía Patológica, Hospital Universitario La Ribera. Alzira, Spain.

^7^Department of Pathology. University Hospital Virgen del Rocío. Sevilla, Spain.

^8^Pathology Department, Arnau de Vilanova University Hospital. Lleida, Spain.

^9^Pathological Anatomy Laboratory. Casa Sollievo della Sofferenza. San Giovanni Rotondo (FG), Italy

^10^Department of Pathology, Hospital Universitario La Paz, Molecular Pathology and Therapeutic Targets Group, Hospital La Paz Insitute of research (IdiPAZ), Center for Biomedical Research in the Cancer Network (CIBERONC), Instituto de Salud Carlos III, Faculty of Medicine, Universidad Autónoma de Madrid. Madrid, Spain.

**Corresponding author:**

Laia Bernet-Vegué

E-mail: [mebernet@riberasalud.es](mailto:mebernet@riberasalud.es)

# Supplementary Methods

- **Total tumor load** was defined as the total sum of CK19 mRNA copies in all positive SLN, and TTL cut-offs were defined by Peg et al [18].
- **Disease free survival** was defined as the time from BC surgery (and SLNB) without evidence of disease (i.e., no progression).
- The **prognostic scoring system** to estimate 10-year DFS was based on NEOVATTL criteria and is described on Table S1.
- **Miller-Payne grading system** is an independent predictor of DFS widely accepted and frequently used. It consists of 5 grades where 5 is a pathological complete response in breast and 1–4 are partial pathological responses according to tumor reduction ratio (Table S2) [22, 23]. Ki-67 is also a prognostic factor for DFS, and its levels are classified as low (<10%), intermediate (10–19%), and high (>20%) ^1-5^.

1. Peg V, Sansano I, Vieites B, et al (2017) Role of total tumour load of sentinel lymph node on survival in early breast cancer patients. The Breast 33:8–13. <https://doi.org/10.1016/j.breast.2017.02.011>
2. Wang W, Liu Y, Zhang H, et al (2021) Prognostic value of residual cancer burden and Miller-Payne system after neoadjuvant chemotherapy for breast cancer. Gland Surg 10:3211–3221. https://doi.org/10.21037/gs-21-608
3. Hall PS, Ironside A, Tramonti G, et al (2022) Abstract P1-02-04: Modified Miller-Payne score as a pragmatic and efficient alternative to Residual Cancer Burden. Cancer Res 82:P1-02-04-P1-02–04. https://doi.org/10.1158/1538-7445.SABCS21-P1-02-04
4. Brown J, Scardo S, Method M, et al (2022) A real-world retrospective study of the use of Ki-67 testing and treatment patterns in patients with HR+, HER2− early breast cancer in the United States. BMC Cancer 22:502. https://doi.org/10.1186/s12885-022-09557-6
5. Panal Cusati M, Herrera de la Muela M, Hardisson Hernaez D, et al (2014) Correlación entre la expresión de Ki67 con factores clásicos pronósticos y predictivos en el cáncer de mama precoz. Revista de Senología y Patología Mamaria 27:163–169. https://doi.org/10.1016/j.senol.2014.07.005

# Supplementary Tables

| **Table S1**. Prognostic scoring system to estimate 10-year DFS based on NEOVATTL criteria | |
| --- | --- |
| Score 1 | TTL of < 25,000 copies/μL, Ki67 of ≤ 20% or Miller-Payne grade of 5 |
| Score 2 | TTL of ≥ 25,000 copies/μL, Ki67 > 20% or Miller-Payne grade of 3 or 4 |
| Score 3 | TTL of ≥ 25,000 copies/μL, Ki67 > 20% or Miller-Payne grade of 1 or 2 |
| Score 4 | TTL of ≥ 25,000 copies/μL and a Ki67 of > 20% |

| **Table S2**. Miller-Payne grading system | |
| --- | --- |
| Grade 1 | No change or some alteration to individual malignant cells but no reduction in overall cellularity. |
| Grade 2 | A minor loss of tumor cells but overall cellularity still high; up to 30% loss. |
| Grade 3 | Between an estimated 30% and 90% reduction in tumor cells. |
| Grade 4 | A marked disappearance of tumor cells such that only small clusters or widely dispersed individual cells remain; more than 90% loss of tumor cells. |
| Grade 5 | No malignant cells identifiable in sections from the site of the tumor; only vascular fibroelastic stroma remains often containing macrophages. However, ductal carcinoma in situ may be present. |

| **Table S3.** Pathologic tumor characteristics before and after NAST, n (%) | | | | |
| --- | --- | --- | --- | --- |
|  | **Total** | **NeovaTTL** | **NeovaTTL Validation** | ***P*-value** |
| **Tumor size,** n | 560 | 314 | 246 |  |
| ≤20mm | 401 (71.6) | 206 (65.6) | 195 (79.3) | 0.0005^(1)^ |
| >20mm | 159 (28.4) | 108 (34.4) | 51 (20.7) |  |
| **Histological type (WHO)** | | | | |
| **Pre NAST,** n | 566 | 312 | 254 |  |
| Ductal carcinoma (without NOS) | 493 (87.2) | 262 (84.0) | 231 (91) | < 0.0001^(2)^ |
| Infiltrating lobular carcinoma | 14 (2.5) | 0 (0.0) | 14 (5.5) |  |
| Metaplastic carcinoma | 2 (0.4) | 0 (0.0) | 2 (0.8) |  |
| Micropapillary carcinoma | 2 (0.4) | 0 (0.0) | 2 (0.8) |  |
| Mucinous carcinoma | 2 (0.4) | 0 (0.0) | 2 (0.8) |  |
| Papillary carcinoma | 1 (0.2) | 0 (0.0) | 1 (0.4) |  |
| Medullary carcinoma | 1 (0.2) | 0 (0.0) | 1 (0.4) |  |
| Other | 51 (9.0) | 50 (16.0) | 1 (0.4) |  |
| **Post NAST,** n | 539 | 312 | 227 |  |
| Adenosis | 1 (0.2) | 0 (0.0) | 1 (0.4) | < 0.0001^(2)^ |
| Ductal carcinoma (including NOS) | 350 (65) | 182 (58.3) | 168 (74.2) |  |
| Absence of infiltrating carcinoma | 1 (0.2) | 0 (0.0) | 1 (0.4) |  |
| Metaplastic carcinoma | 2 (0.4) | 0 (0.0) | 2 (0.9) |  |
| Micropapillary carcinoma | 1 (0.2) | 0 (0.0) | 1 (0.4) |  |
| Mucinous carcinoma | 3 (0.6) | 0 (0.0) | 3 (1.3) |  |
| Medullary carcinoma | 1 (0.2) | 0 (0.0) | 1 (0.4) |  |
| In situ lobular carcinoma | 1 (0.2) | 0 (0.0) | 1 (0.4) |  |
| Infiltrating lobular carcinoma | 16 (3.0) | 0 (0.0) | 16 (7.0) |  |
| Infiltrating mucinous carcinoma | 1 (0.2) | 0 (0.0) | 1 (0.4) |  |
| Fibrosis | 2 (0.4) | 0 (0.0) | 2 (0.9) |  |
| Sclerosis | 1 (0.2) | 0 (0.0) | 1 (0.4) |  |
| Fibrosclerosis | 1 (0.2) | 0 (0.0) | 1 (0.4) |  |
| Fibrosis | 4 (0.7) | 0 (0.0) | 4 (1.8) |  |
| Neuroendocrine | 1 (0.2) | 0 (0.0) | 1 (0.4) |  |
| Papillary solid infiltrating | 2 (0.4) | 0 (0.0) | 2 (0.9) |  |
| Tubulolobular | 1 (0.2) | 0 (0.0) | 1 (0.4) |  |
| pCR | 99 (18.4) | 81 (26.0) | 18 (7.9) |  |
| Other | 51 (9.5) | 49 (15.7) | 2 (0.9) |  |
| **Tumor grade** | | | | |
| **Pre NAST,** n | 515 | 296 | 219 |  |
| G1 | 63 (12.2) | 36 (12.2) | 27 (12.3) | 1.0000^(1)^ |
| G2 and G3 | 452 (87.8) | 260 (87.8) | 192 (87.7) |  |
| **Post NAST,** n | 506 | 307 | 199 |  |
| G1 | 110 (21.7) | 62 (20.2) | 48 (24.1) | < 0.0001^(2)^ |
| G2 and G3 | 305 (60.3) | 164 (53.4) | 141 (70.9) |  |
| pCR | 91 (18.0) | 81 (26.4) | 10 (5.0) |  |
| **ER positive cells** | | | | |
| **Pre NAST,** n | 561 | 312 | 249 |  |
| Negative | 151 (26.9) | 84 (26.9) | 67 (26.9) | 1.0000^(1)^ |
| Positive ( ≥1%) | 410 (73.1) | 228 (73.1) | 182 (73.1) |  |
| **Post NAST,** n | 229 | 111 | 118 |  |
| Negative | 49 (21.4) | 21 (18.9) | 28 (23.7) | 0.4220^(1)^ |
| Positive ( ≥1%) | 180 (78.6) | 90 (81.1) | 90 (76.3) |  |
| **PR cells (%)** | | | | |
| **Pre NAST,** n | 548 | 306 | 242 |  |
| Negative | 210 (38.3) | 116 (37.9) | 94 (38.8) | 0.8597^(1)^ |
| Positive ( ≥1%) | 338 (61.7) | 190 (62.1) | 148 (61.2) |  |
| **PR (%)** | | | | |
| **Post NAST,** n | 229 | 111 | 118 |  |
| Negative | 108 (47.2) | 55 (49.5) | 53 (44.9) | 0.5097^(1)^ |
| Positive ( ≥1%) | 121 (52.8) | 56 (50.5) | 65 (55.1) |  |
| **HER2** | | | | |
| **Pre NAST,** n | 566 | 314 | 252 |  |
| Negative | 415 (73.3) | 233 (74.2) | 182 (72.2) | 0.6329^(1)^ |
| Positive | 151 (26.7) | 81 (25.8) | 70 (27.8) |  |
| **Post NAST,** n | 427 | 293 | 134 |  |
| Negative | 396 (92.7) | 278 (94.9) | 118 (88.1) | 0.0156^(1)^ |
| Positive | 31 (7.3) | 15 (5.1) | 16 (11.9) |  |
| **Ki67 (%)** | | | | |
| **Pre NAST,** n | 571 | 311 | 260 |  |
| ≤20% | 239 (41.9) | 126 (40.5) | 113 (43.5) | 0.4963^(1)^ |
| >20% | 332 (58.1) | 185 (59.5) | 147 (56.5) |  |
| **Post NAST,** n | 229 | 107 | 122 |  |
| ≤20% | 164 (71.6) | 72 (67.3) | 92 (75.4) | 0.1885^(1)^ |
| >20% | 65 (28.4) | 35 (32.7) | 30 (24.6) |  |
| **HER2 and Ki67 combination** | | | | |
| **Pre NAST,** n | 568 | 314 | 254 |  |
| HER2 (+) or  Ki67>20% | 374 (65.8) | 204 (65.0) | 170 (66.9) | 0.6568^(1)^ |
| HER2 (-) and  Ki67≤20% | 194 (34.2) | 110 (35.0) | 84 (33.1) |  |

NAST, neoadjuvant systemic treatment; WHO, world health organization; pCR, pathologic complete response; G1, G2, G3 grade 1,2,3; NA, not applicable; NOS, invasive breast carcinoma, not otherwise specified; ER, estrogen receptor; PR, progesterone receptor; LUM A, luminal A; LUM B, luminal B HER2, human epidermal growth factor receptor 2; TN, triple negative.

^(1)^ Fisher’s exact test comparing both studies; ^(2)^ Pearson chi-squared test comparing both studies

| **Table S4.** Multivariate Cox validation with HR adjustment analysis, (n =231) | | | | | | |
| --- | --- | --- | --- | --- | --- | --- |
|  | | **HR** | **95% CI low** | **95% CI up** | ***P*-value** | **LRT**  ***P*-value** |
| TTL > 25000 vs TTL ≤ 25000 | | 1.029 | 0.470 | 2.255 | 0.9424 | 0.226 |
| Ki67 PRE* >20% vs ≤ 20% | | 0.484 | 0.245 | 0.959 | 0.0374* |  |
| Miller Payne (REV)** | | 0.931 | 0.691 | 1.255 | 0.6394 |  |
| Score |  | 0.82 | 0.601 | 1.12 | 0.2130 | 0.204 |

HR, hazard ratio; 95%CI, 95% confidence interval; Low, lower bound; Up, Upper bound; LRT, Log Ratio Test; TTL, total tumor load

*Before neoadjuvant systemic therapy

**Revised version of Miller-Payne

| **Table S5**. ROC curve analysis for TTL and non-SLN involvement in the NEOVATTL and validation cohorts | | |
| --- | --- | --- |
| **TTL values (copies/µL)** | **NEOVATTL (%)** | **NEOVATTL Validation (%)** |
| **>250** |  |  |
| Sensitivity | 95.7 | 80.6 |
| Specificity | 72.4 | 68.7 |
| Negative predictive value | 99.0 | 95.5 |
| Positive predictive value | 37.3 | 30.2 |
| **>5000** |  |  |
| Sensitivity | 63.0 | 69.4 |
| Specificity | 86.2 | 90.7 |
| Negative predictive value | 93.1 | 94.6 |
| Positive predictive value | 43.9 | 55.6 |
| **>15000** |  |  |
| Sensitivity | 43.5 | 58.3 |
| Specificity | 92.9 | 93.0 |
| Negative predictive value | 90.5 | 93.0 |
| Positive predictive value | 51.3 | 58.3 |

# Supplementary Figures


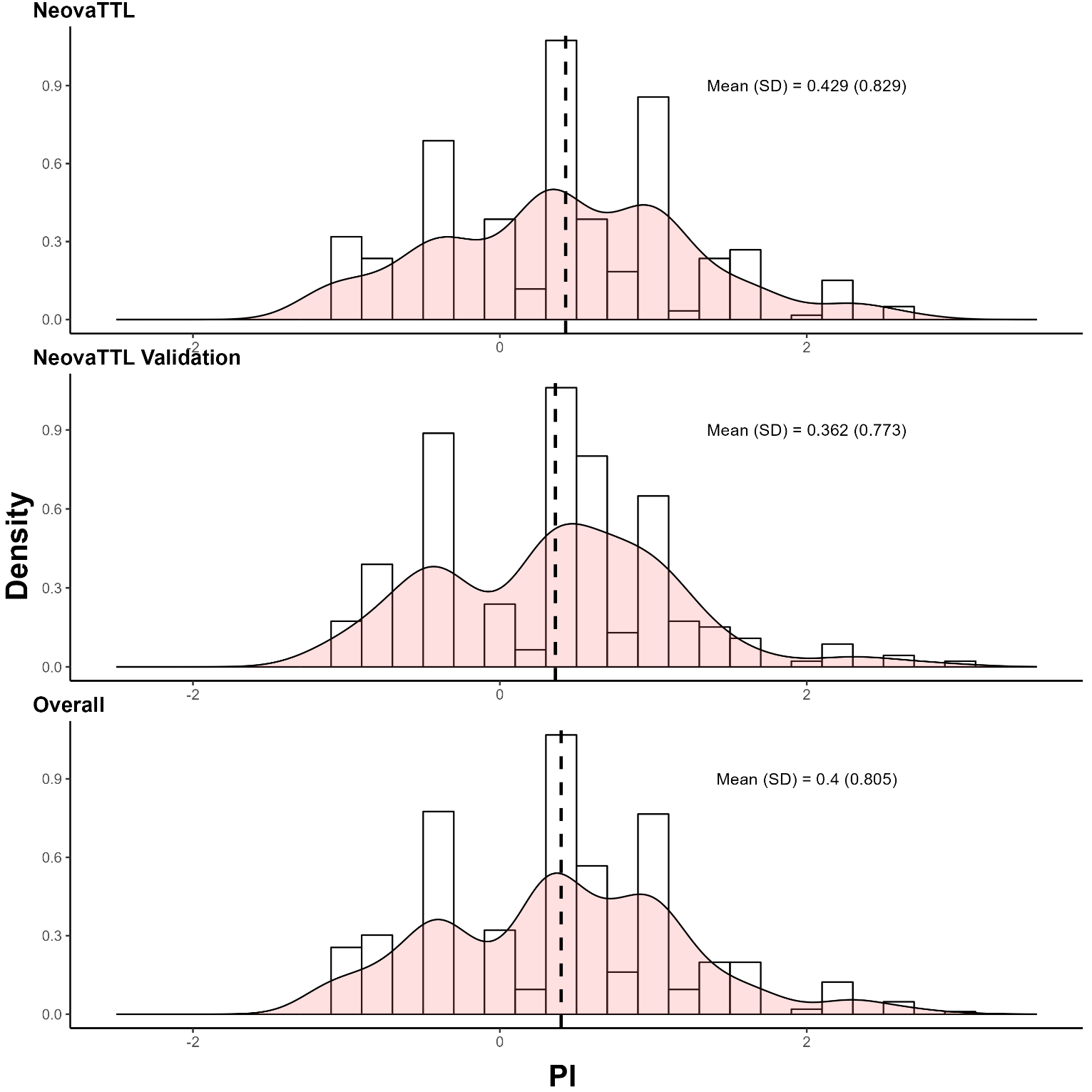


**Figure S1.** PI Distribution for the validation of the Multivariate Cox model (TTL, Ki67, Miller-Payne). A) NEOVATTL study B) NEOVATLL Validation C) Overall

PI, prognostic index; TTL, total tumor load; SD, standard deviation.


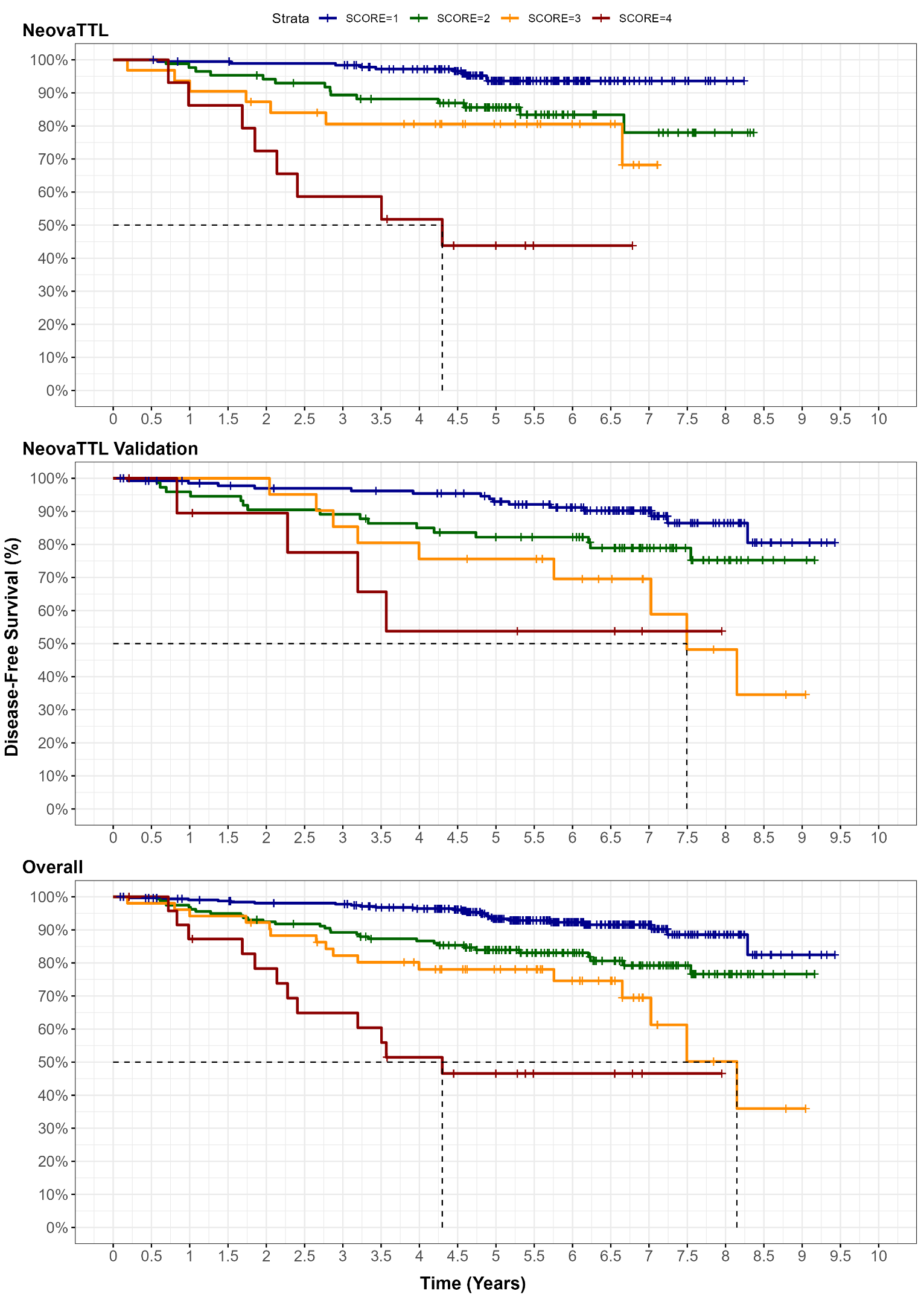


**Figure S2**. Kaplan-Meier estimates of disease-free survival according to scores. A) NEOVATTL study B) NEOVATLL Validation C) Overall


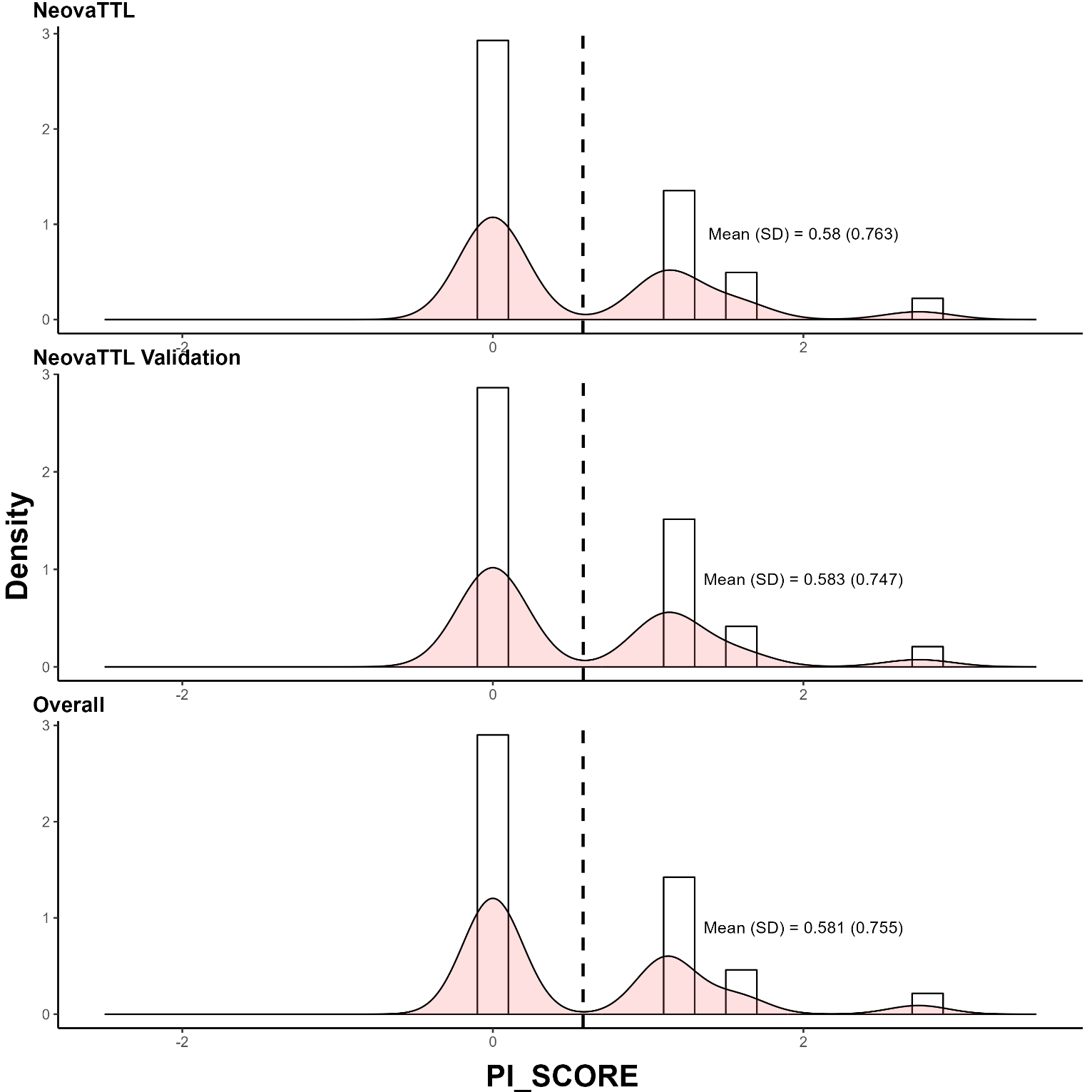


**Figure S3.** PI Distribution for the validation of the Multivariate Cox model (Scores). A) NEOVATTL study B) NEOVATLL Validation C) Overall

PI, prognostic index; SD, standard deviation.
